# ASO Author Reflections: Prediction of Morbidity and Mortality After Esophagectomy: A Systematic Review

**DOI:** 10.1245/s10434-024-15089-z

**Published:** 2024-03-07

**Authors:** M. P. van Nieuw Amerongen, H. J. de Grooth, G. L. Veerman, K. A. Ziesemer, M. I. van Berge Henegouwen, P. R. Tuinman

**Affiliations:** 1 Department of Adult Intensive Care Medicine, Amsterdam UMC (VUmc), Amsterdam, The Netherlands; 2 Medical Library, Vrije Universiteit, Amsterdam, The Netherlands; 3 Department of Surgery, Amsterdam UMC, Location University of Amsterdam, Amsterdam, The Netherlands; 4 Cancer Center Amsterdam, Cancer Treatment and Quality of Life, Amsterdam, The Netherlands; 5 Amsterdam Institute for Immunology and Infectious Diseases, Amsterdam, The Netherlands

## Past

Treatment of esophageal malignancies with surgical esophagectomy is a high-risk procedure with a complication rate of up to 60%.^1^ Predicting the risk of complications could have several potentially important health care benefits. A large number of preoperative prediction models on morbidity and mortality after esophagectomy have been developed in recent years, but their usefulness has not yet been assessed systematically.

## Present

We conducted a systematic review that included 22 studies with 33 different models, of which 18 models were newly developed.^2^ The prognostic accuracy of models differed between 0.51 and 0.85. For most models, the required variables are readily available. Many studies showed a high risk of bias and none of the prediction models were rigorously validated. Two prediction models for mortality and one model for pulmonary complications have the potential to be developed further.^3–5^ None of the models were ready for clinical implementation.

## Future

This review shows that several models are promising but need to be further developed. If improved, the models could provide significant benefits for patients with esophageal cancer. Early identification of high-risk patients allows for informed decision making, personalized preventive measures targeting modifiable risk factors, and closer monitoring of those at the highest risk for timely complication detection, potentially avoiding non-cost-effective interventions for the entire population. However, future models do need to be robustly developed and validated in other populations.
